# A non-mitotic role for Aurora kinase A as a direct activator of cell migration upon interaction with PLD, FAK and Src

**DOI:** 10.1242/jcs.157339

**Published:** 2015-02-01

**Authors:** Madhu Mahankali, Karen M. Henkels, Francis Speranza, Julian Gomez-Cambronero

**Affiliations:** Wright State University School of Medicine, Department of Biochemistry and Molecular Biology, Dayton, OH 45435, USA

**Keywords:** Aurora kinase, Tubulin polymerization, Cell migration, Cell signaling, Leukocytes, Phosphatidic acid

## Abstract

Timely activation of Aurora kinase A (AURA, also known as AURKA) is vital for centrosome formation and the progression of mitosis. Nonetheless, it is still unclear if and when other cellular functions are activated by AURA. We report here that Src phosphorylates and activates AURA at T288, and AURA also activates focal adhesion kinase (FAK, also known as PTK2), leading to initiation of cell movement. An additional and new way by which AURA is regulated, is by phospholipase D2 (PLD2), which causes AURA activation. In addition, AURA phosphorylates PLD, so both proteins engage in a positive reinforcement loop. AURA and PLD2 form a protein–protein complex and colocalize to cytoplasmic regions in cells. The reason why PLD activates AURA is because of the production of phosphatidic acid by the lipase, which binds directly to AURA, with the region E^171^–E^211^ projected to be a phosphatidic-acid-binding pocket. Furthermore, this direct interaction with phosphatidic acid enhances tubulin polymerization and cooperates synergistically with AURA, FAK and Src in yielding a fully effectual cellular migration. Thus, Src and FAK, and PLD and phosphatidic acid are new upstream regulators of AURA that mediate its role in the non-mitotic cellular function of cell migration.

## INTRODUCTION

Mitosis is mediated by phosphorylation and dephosphorylation of specific substrates by many different protein kinases, such as the Polo-like kinases (PLKs), the cyclin-dependent kinases (CDKs), the never in mitosis gene a kinase (NIMA) and the aurora kinases (AURKs). The Aurora kinase family (comprising Aurora A, B and C) is a group of cell-cycle-regulated enzymes that controls several aspects of cell division in mammalian cells (Vader and Lens, 2008).

The Aurora kinase A isoform (AURA, also known as AURKA and STK15), is a 403-amino-acid 46 kDa protein that promotes microtubule nucleation around the chromatin, through phosphorylation of NEDD1, and functional spindle assembly in mitotic cells (Yang et al., 2000; Pinyol et al., 2013). The AURA-encoding gene is located on chromosome 20q13.2-q13.3, a region frequently amplified in human malignancies, and AURA is considered to be a bona fide oncogene (Bar-Shira et al., 2002). AURA is localized to centrosomes during G2 and mitosis on the mitotic spindle (Vader and Lens, 2008). Additionally, AURA is associated with microtubules in the cytoplasm (Vader and Lens, 2008).

Several upstream regulators of AURA have been identified, including Ajuba and targeting protein for Xklp2 (TPX2) and cyclin-dependent kinase 1 (CDK1), which are capable of promoting the phosphorylation and kinase activity of AURA (Hirota et al., 2003; Eckerdt et al., 2009; Jantscher et al., 2011). The heterodimeric protein–protein complex of AURA–Ajuba (similar to the Cdk1–cyclinB1 complex) can be detected prior to mitosis. During specific phases of the cell cycle, AURA function is regulated by phosphorylation at the conserved T288 residue within the activation loop of its catalytic domain (Walter et al., 2000; Ferrari et al., 2005). The result of this phosphorylation is a significant increase in its enzymatic activity, but other possible ways of activating AURA are currently under intense study, as it is unclear what processes lead to the activation of AURA alone or in complex with some other factor or protein.

The localization of AURA to centrosomes at the beginning of the mitotic spindle formation has received much attention. However, it has become clear that low levels of AURA still remain in other phases of the cycle and can be detected by immunofluorescence as diffuse localization. The fact that AURA exists outside the M phase could indicate that it still modulates other cellular functions. As AURA fully participates in the pulling of chromosomes attracting them to the bipolar centromeres, we reasoned that AURA could perhaps have a motility-related function in the cytoplasm during interphase. We have found that, indeed, AURA is implicated in cell migration along with Src and FAK and, unexpectedly, is regulated by a lipase, phospholipase D2 (PLD2). Additionally, phosphatidic acid, a key intracellular signaling phospholipid, binds to and positively affects the activity of AURA leading to tubulin polymerization and cell motility.

## RESULTS

### AURA is essential for enhanced cell migration

**Fig. 1.**
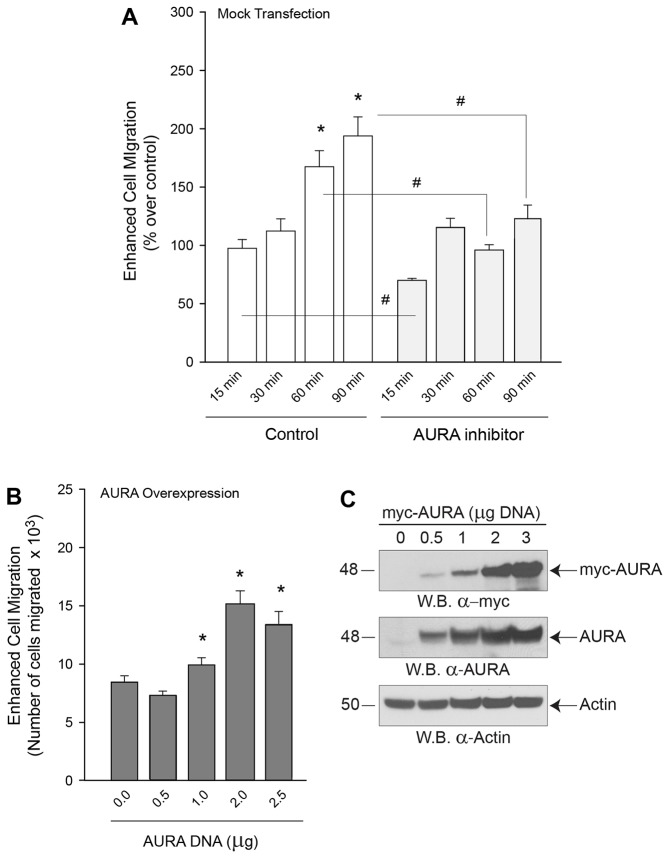
**AURA induces enhanced cell migration.** (A) COS-7 fibroblasts had enhanced motility in Transwell settings upon induction with EGF (which acts here as a ‘motogen’), and this was inhibited by the AURA inhibitor MLN 8237 (20 nM). (B) COS-7 cells were either mock-transfected or transfected with the indicated amount of AURA plasmid DNA. At 2 days post-transfection enhanced cell migration, as assessed using Transwells, was significantly increased by AURA overexpression. Experiments were performed in triplicate and are expressed as the mean±s.e.m. number of cells that migrated (percentage of control). *P<0.05 increases and ^#^*P*<0.05 decreases between samples and controls. (C) Positive controls showing the presence of overexpressed and endogenous AURA as detected by western blotting (W.B.). Actin is shown as an equal protein loading control.

In Fig. 1A, enhanced cell migration of COS-7 fibroblasts towards 3 nM EGF was mediated by endogenous AURA, as the presence of the AURA-specific small-molecule inhibitor, AM-8237 (30 nM) significantly reduced migration. Additionally, enhanced cell migration in the presence of increasing concentrations of overexpressed AURA (Fig. 1B) resulted in an increase of cell migration of ∼2.3-fold compared with mock-treated cells. Fig. 1C shows western blot analyses indicating expression of endogenous, as well as overexpressed AURA.

### Silencing AURA reduces cell migration by modulating tubulin

**Fig. 2.**
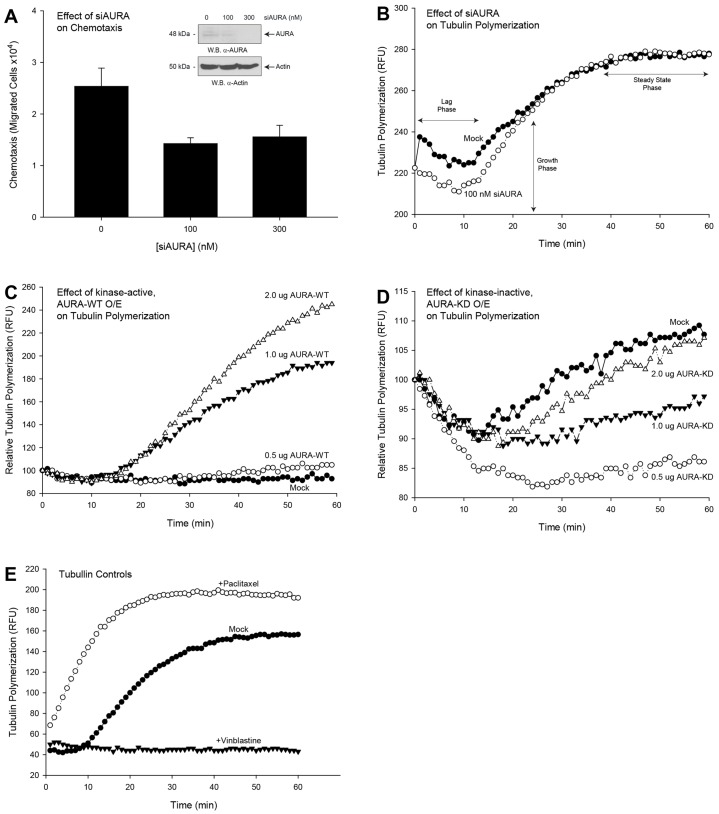
**Silencing AURA reduced cell migration by modulating tubulin.** Using COS-7 cell lysates that were silenced with up to 300 nM siRNA specific for AURA for 3 days, samples were prepared for SDS-PAGE and subsequent western blot analysis or for tubulin polymerization assays. (A inset) Western blot analyses of COS-7 cell lysates that were transfected with increasing siRNA specific for AURA (siAURA). (A) Enhanced cell migration is decreased in cells treated with increasing amounts siAURA. Experiments were performed in triplicate and are expressed as the mean±s.e.m. number of cells that migrated (percentage of control) *P<0.05 increases and ^#^*P*<0.05 decreases between samples and controls. (B–D) Tubulin polymerization of cells that were either silenced for AURA (siAURA) or that overexpressed (O/E) AURA. (B) Tubulin polymerization was reduced early in the polymerization reaction and not at later times as a result of silencing AURA as compared to non-silenced samples. (C) Overexpressed wild-type AURA (AURA-WT) had a positive effect on tubulin polymerization compared to mock cells. (D) The positive effect on tubulin polymerization was reversed when the kinase-inactive mutant AURA-D274A (AURA-KD) was overexpressed. (E) Tubulin polymerization positive (taxol) and negative (BSA) controls. Experiments were performed in triplicate and are expressed as the mean relative number of fluorescence units (RFU).

Having shown that overexpression of AURA enhanced cell migration, we next wanted to determine the effect of silencing AURA on enhanced cell migration. As shown in the Fig. 2A inset, western blot analyses confirm downregulation of AURA using small interfering RNA (siRNA) specific for AURA. Results shown in Fig. 2A indicate that, as a result of AURA silencing, enhanced cell migration was significantly reduced in a dose-dependent manner. In addition to AURA being important to the process of chemotaxis, AURA also contributes to another important physiological process, microtubule organization, which is essential to cell movement (Ballestrem et al., 2000; Yao et al., 2004). Therefore, we next determined whether AURA regulated cell migration by modulating tubulin polymerization. Using COS-7 cell lysates that were silenced with up to 300 nM siRNA specific for AURA, tubulin polymerization assays were performed. Interestingly, tubulin polymerization was reduced early in the polymerization reaction and not at later times. We observed a decrease in terms of the lag and growth phases of the polymerization reaction as a result of silencing AURA (Fig. 2B) when compared to non-silenced samples. Furthermore, cells that overexpressed increasing amounts of wild-type AURA had concomitantly increased tubulin polymerization (Fig. 2C), which further confirmed the positive effect of AURA overexpression on cell function. This positive effect was reversed when the kinase-inactive mutant AURA-D274A was overexpressed, which stresses the importance of catalytically active AURA to this process (Fig. 2D). Fig. 2E is representative of the positive (taxol) and negative (BSA) controls for tubulin polymerization, respectively.

### Src contributes to AURA-mediated cell migration

**Fig. 3.**
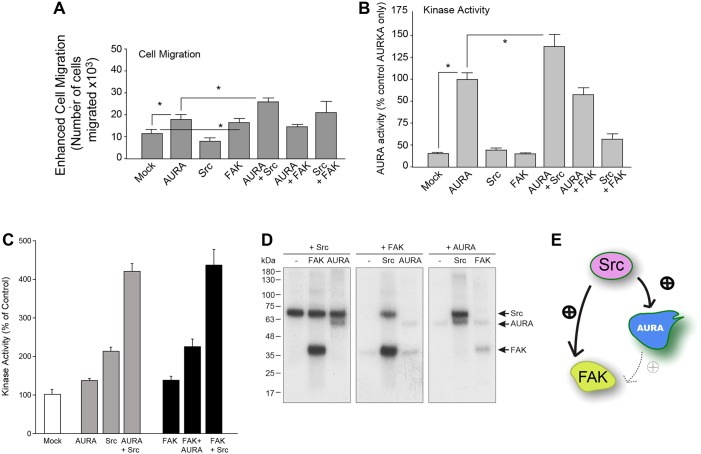
**Src and FAK contribute to AURA-mediated cell migration.** Effect of overexpression of cell motility proteins on AURA-mediated enhanced cell migration (A) and AURA activity (B). (C,D) Phosphorylation states of purified recombinant AURA, FAK and Src. (C) Kinase activities using purified recombinant AURA, FAK and/or Src as phosphorylation substrate for the relevant kinase of interest, which were then used for filter-binding assays and subsequent liquid scintillation spectrometry. Results were quantified as the mean±s.e.m. percentage activity compared to the control sample. *P<0.05 increases and ^#^*P*<0.05 decreases between samples and controls. (D) Phosphorylation in-gel analyses of Src, FAK or AURA in the absence or presence of the other kinase. Recombinant proteins were individually incubated with buffer alone or one of the other kinases in a buffer containing [^32^P]γATP, following which phosphorylation of the kinases was observed by SDS-PAGE and then western blot analyses. (E) Schematic drawing representing activation of upstream Src from the downstream AURA or FAK.

Given the fact that motile proteins like Src and FAK are found at focal adhesions during migration and at the initial steps of cell migration, we hypothesized that either of these two proteins could mediate the increase in cell migration we observed in Fig. 1. Fig. 3 shows that there was an enhanced cell migration (Fig. 3A) and AURA activity (Fig. 3B) of COS-7 epithelial cells upon ectopic overexpression of AURA. It further shows that Src increases the amount of migration when present together with AURA. The results of this line of experimentation indicate that cell migration was maximal with AURA and Src co-overexpression compared to other samples, which was substantiated by the very substantial increase in kinase activity (Fig. 3B, third bar from the right). To better understand the dynamics of these three kinases, we used them in varying combinations with the other kinases of interest in the presence of the relevant reaction components that included radioactive [^32^P]γATP. We then assayed their *in vitro* kinase activities by determining the amount of [^32^P]γATP incorporated. Both the overall level of radiolabel that was incorporated (the overall level of phosphorylation) on the kinases collectively was detected (using a filter-binding assay; Fig. 3C) and the actual individual level of phosphorylation of each kinase in the reaction was detected (using an in-gel analysis; Fig. 3D). The samples shown in Fig. 3D are those derived from Fig. 3C but visualized after SDS-PAGE and transfer to PVDF membranes and subsequent autoradiography. As shown in these two panels, we found that Src strongly phosphorylated both FAK and AURA, whereas FAK phosphorylation of AURA and vice versa led to much lower levels of phosphorylation. We interpret this data to indicate that Src is upstream of both FAK and AURA. A schematic of regulation between these three kinases is shown in Fig. 3E, suggesting that Src was the upstream kinase regulating both FAK and AURA, whereas FAK might be downstream of AURA (as AURA phosphorylation of FAK yielded more incorporation of [^32^P]γATP compared to FAK phosphorylation of AURA).

### PLD2 contributes to AURA-mediated cell migration

**Fig. 4.**
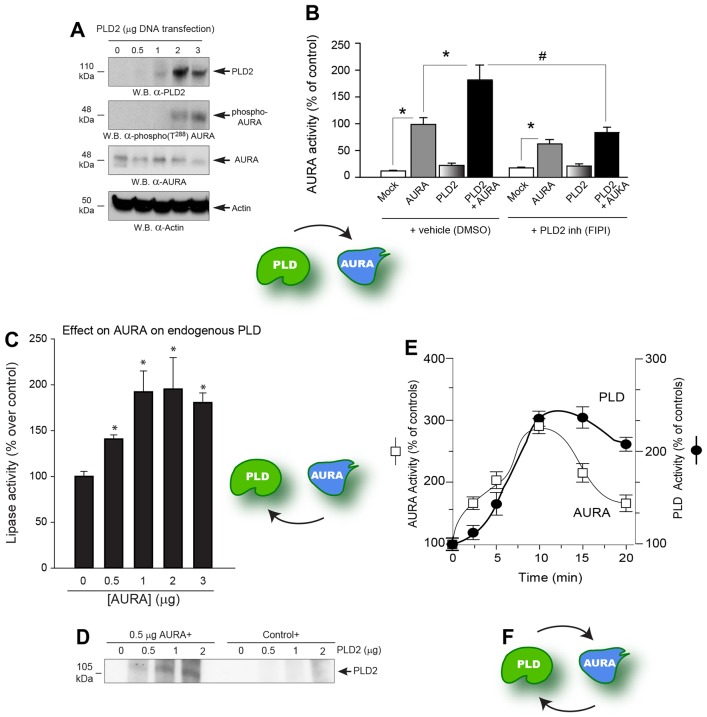
**Reciprocal activation between PLD and AURA.** (A) PLD2 overexpression resulted in AURA phosphorylation. PVDF membranes were probed for Myc-tagged PLD2, phospho-AURA (T288), total AURA or actin using relevant rabbit monoclonal antibodies. (B) PLD2 overexpression increased AURA activity. COS-7 cell lysates that overexpressed Mock, AURA alone, PLD2 alone or both AURA plus PLD2 were incubated in the absence or presence of 300 nM FIPI (PLD2-specific small-molecule inhibitor) and used for a AURA catalytic assay that incorporated [^32^P]γATP on the synthetic AURA-T288 peptide substrate. Experiments were performed in triplicate and are expressed as the mean±s.e.m. percentage of control. *P<0.05 increases and ^#^*P*<0.05 decreases between samples and controls. (C,D) Effects of AURA on PLD. AURA overexpression caused an increase in both PLD activity of COS-7 cell lysates (C) and an increase in the level of phosphorylation of PLD2 when recombinant PLD2 was used as a phosphorylation substrate for recombinant AURA in an *in vitro* kinase assay. (D). (E) Indicates the cellular activity of endogenous PLD or AURA when activated with 3 nM EGF. Anti-AURA immunoprecipitates or anti-PLD immunoprecipitates were used for *in vitro* assays: one that measured the incorporation of [^32^P]γATP to the specific peptide substrate for AURA (white squares) or one that measured phospholipase activity in the presence of PC8 liposomes and [^3^H]butanol (black circles). (F) Indicates the reciprocal activation of phopholipase and kinase.

We found that PLD2 overexpression in COS-7 epithelial cells exerted a concomitant positive effect on AURA phosphorylation at T288 (Fig. 4A), as detected using an antibody specific to this residue on AURA, and also on the autocatalytic activity of AURA as determined by measuring its activity towards a synthetic peptide substrate that mimicked its autophosphorylation site at T288 (Fig. 4B). Moreover, a small-molecule inhibitor of PLD2 activity (FIPI) negated the gain produced by co-overexpression of both PLD2 and AURA on AURA activity. Next, we investigated whether the inverse scenario were true, that is, did AURA exert a positive effect on PLD2 activity. As shown in Fig. 4C, AURA exerted a statistically significant positive effect on PLD lipase activity when cell lysates that overexpressed AURA were used for the PLD *in vitro* assay, which was manifested as a >2-fold increase in total lipase activity compared to the negative control sample.

Using purified, baculoviral PLD2 protein as a full-length protein phosphorylation substrate for purified recombinant AURA in an *in vitro* kinase assay measuring incorporation of [^32^P]γATP onto PLD2 by subsequent autoradiography of the resulting PVDF membrane, we detected a phosphorylated PLD2 (phospho-PLD2) band, as the result of AURA action, which was present at the expected molecular mass of wild-type PLD2 (∼105 kDa) (Fig. 4D). This result indicated that PLD2 was phosphorylated by AURA. Further, similar cell samples that were stimulated with 3 nM EGF for several periods of time and then immunoprecipitated with either anti-PLD or anti-AURA antibodies indicate that both activities run somewhat in parallel (Fig. 4E), as the result of a continuous activation loop between PLD2 and AURA that has been described herein for the first time (Fig. 4F).

### PLD2 and AURA colocalize and form a protein–protein heterodimer

**Fig. 5.**
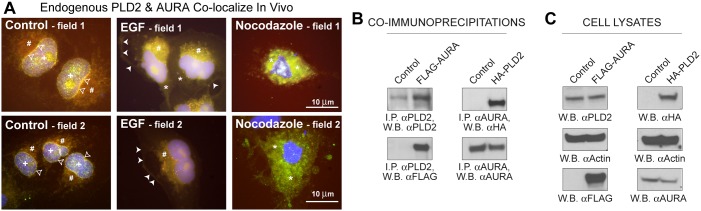
**PLD2 and AURA colocalize and form a protein–protein heterodimer.** (A) Merged immunofluorescence microscopic images of COS-7 cells stained for endogenous AURA (red), endogenous PLD2 (green) or both endogenous proteins together (merged yellow fluorescence) using antibodies specific to each protein (TRITC labeling for AURA, FITC labeling for PLD2 and blue nuclei visualized using DAPI). Both AURA and PLD2 were found to be colocalized at the nucleus (+ symbols), nuclear membrane (black arrowheads) and Golgi (# symbols) in the absence of stimulation (left panels). Following 3 nM EGF stimulation for 15 min (middle panels), both AURA and PLD2 were found to be colocalized at non-nuclear regions of the cells, which included the plasma membrane (white arrowheads) and cytoplasm (* symbols) and suggests a shift in the cell to that of a migratory phenotype. Results also suggest a very close cytoplasmic localization occurred between the two proteins in the cells treated with nocodazole (right panels). This might be due to the fact that upon inhibition of mitosis, AURA is localized in the cytosol rather than in the proximity of the nucleus. Shown are two representative fields of images for each sample among 20 for each sample that were visualized. Scale bars: 10 µm. (B,C) COS-7 cells were either mock-transfected or transfected with plasmids expressing FLAG–AURA or HA–PLD2 and at 2 days post-transfection were used for co-immunoprecipitation (I.P.). (B) When AURA was immunoprecipitated, overexpressed HA-tagged PLD2 was pulled down as shown when samples were probed with anti-HA antibodies recognizing PLD2 (top right panel). When PLD2 was immunoprecipitated using antibodies specific to the phospholipase, overexpressed FLAG-tagged AURA was pulled down when samples were probed with anti-FLAG antibodies recognizing AURA (bottom left panel). Positive controls for both PLD2 and AURA are shown in the top left and bottom right panels, respectively. (C) Protein inputs and equal protein loading controls for each set of samples shown in the co-immunoprecipitations in B.

The fact that a kinase and a phospholipase activate each other indicates that both proteins could be situated proximally to each other. To determine the cellular localization patterns of PLD2 and AURA within the cell, immunofluorescence microscopy was performed using COS-7 cells that were untreated or treated with either 3 nM EGF or 1 µM nocodazole (a well-characterized mitosis inhibitor). As we are interested in establishing whether AURA and PLD2 colocalize in the cell, we focused only on cells that showed co-fluorescence of both signaling proteins. Fig. 5 depicts the immunofluorescence images of cells stained for both endogenous AURA and endogenous PLD2. As shown in the immunofluorescence data presented in Fig. 5A, in the absence of any stimulation (Fig. 5A, left panels), both AURA and PLD2 were found to be colocalized at the nucleus (+ symbols), nuclear membrane (open arrowheads) and Golgi (# symbols). Following EGF stimulation (Fig. 5A, middle panels), both AURA and PLD2 were found to be colocalized at non-nuclear regions of the cells, which included the plasma membrane (white arrowheads) and cytoplasm (* symbols) and suggests a shift in the cell to that of a migratory phenotype. Results also suggest that a very close cytoplasmic localization (* symbols) occurred between the two proteins in the cells treated with nocodazole (Fig. 5A, right panels). This might be because, upon inhibition of mitosis, AURA is localized primarily in the cytosol rather than in the proximity of the nucleus, as is PLD2. Overexpression of AURA in *Xenopus* cells has been documented to lead to its localization at centrosomes, the nucleus and the cytoplasm (Rannou et al., 2008). Therefore, our results regarding AURA cellular localization are in line with this earlier report. Additionally, we now document that a phospholipase, specifically PLD2, is also found to be localized to similar locations in the cells as that of AURA, especially in cells stimulated with a chemoattractant known to induce cells to migrate.

Fig. 5B further confirms the existence of a protein–protein interaction between AURA and PLD2 as determined by co-immunoprecipitations that were performed with endogenous proteins (either AURA or PLD2, respectively) and then looking for the companion overexpressed protein (either HA-tagged PLD2 or FLAG-tagged AURA, respectively). As shown, when AURA was immunoprecipitated using antibodies specific to the kinase, overexpressed HA-tagged PLD2 was pulled down (Fig. 5B, top right panel). The inverse interaction was also tested. When PLD2 was immunoprecipitated using antibodies specific to the phospholipase, overexpressed FLAG-tagged AURA was pulled down (Fig. 5B, bottom left panel). Positive controls for both PLD2 and AURA are shown in the top left and bottom right panels, respectively. Fig. 5C represents the protein inputs and equal protein loading controls for each set of samples shown in the co-immunoprecipitations. Overall, data from Fig. 5 suggests that AURA and PLD2 interact in the cell in a manner that is mediated by EGF signaling.

### The product of PLD action, phosphatidic acid, is also involved in AURA activation and changes tubulin dynamics and helps AURA promote cell migration

**Fig. 6.**
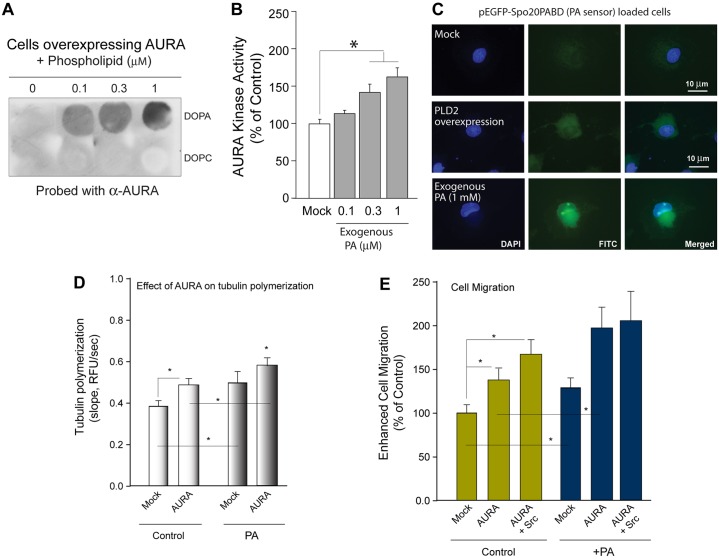
**Phosphatidic acid binds to and activates AURA and positively affects tubulin polymerization.** (A) Protein–lipid overlay assays using 1,2-dioleoyl-sn-glycero-3-phosphocholine (DOPA) and lysates from cells overexpressing AURA. DOPC was used as a negative control. (B) Positive effect of increasing concentrations of phosphatidic acid (PA) on catalytic activity of recombinant purified AURA. Experiments were performed in triplicate and are expressed as the mean±s.e.m. percentage of control. *P<0.05 increases between samples and controls. (C) Immunofluorescence microscopy of positive controls that indicate that phosphatidic acid can be effectively visualized inside cells. COS-7 cells were transfected with the pEGFP-Spo20PABD phosphatidic acid sensor in the absence or presence of PLD2 overexpression and cultured for 2 days or in the presence of exogenous phosphatidic acid (1 µM, 10 min), fixed and stained with DAPI to visualize nuclei (blue). Shown are representative fields of images among 20 that were visualized. Scale bars: 10 µm. (D) Measurement of tubulin polymerization in the presence or absence of recombinant purified AURA. (E) Enhanced cell migration was measured using Transwells 2 days post-transfection for cells that overexpressed AURA alone or in combination with Src in the absence or presence of 300 nM phosphatidic acid. *P<0.05 increases between samples and controls.

Although the immunofluorescence microscopy and immunoprecipitation data shown in Fig. 5 indicate that AURA and PLD2 colocalize in the cell and form a protein–protein interaction that positively influenced AURA activity, we did not know whether this interaction was mediated by the product of the PLD reaction, phosphatidic acid. As shown in Fig. 6A, overexpressed AURA from COS-7 cells lysates bound to exogenous phosphatidic acid (but not the negative control, phosphatidylcholine) through a protein–lipid interaction detected using a PVDF membrane and subsequent incubation with an antibody specific for AURA. Additionally, exogenous phosphatidic acid added to the cells was able to augment the activity of AURA (Fig. 6B) and could also be detected inside similarly treated cells using a phosphatidic acid biosensor (Fig. 6C).

As shown in Fig. 2, it is clear that AURA had a significant positive effect on tubulin polymerization. Next, we determined whether phosphatidic acid had any effect on AURA-mediated tubulin polymerization. Fig. 6D indicates that phosphatidic acid provided a gain of function to AURA-led tubulin polymerization. Additionally, these results are in agreement with cell migration results, where AURA gains functionality in the presence of Src and phosphatidic acid (Fig. 6E).

### A putative phosphatidic-acid-binding region in AURA

**Fig. 7.**
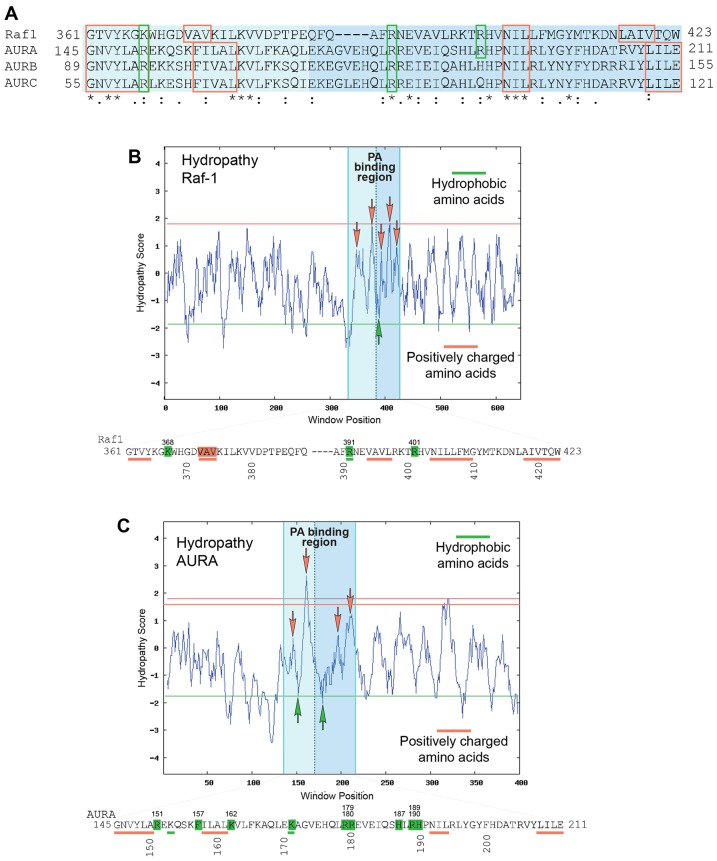
**AURA shares homology with a unique region on Raf-1 that binds phosphatidic acid.** (A) ClustalW multiple alignment of Aurora kinases A, B and C and Raf-1 shows a substantial similarity of the three AURA proteins with the putative phosphatidic acid (PA)-binding site of Raf-1. The homologous region in Raf-1 (B) and AURA (C) showed hydropathy fluctuations between positively charged amino acids such as arginine, histidine and lysine residues, on one hand, and hydrophobic amino acids such as leucine and valine residues, on the other hand, that could very well accommodate both the negative polar head and the hydrophobic side chains of phosphatidic acid, respectively. Kyte-Doolittle hydropathy plots for Raf-1 (B) and AURA (C). Query statistics were window size = 9; start position-1; end position = 403; query length = 403; effective length = 395.

Given that Fig. 6 indicates that phosphatidic acid binds to AURA, we next determined the putative phosphatidic-acid-binding region on AURA. As Raf-1 is an well-known binding partner of phosphatidic acid (Ghosh et al., 1996), multiple sequence alignments were performed with AURA, Aurora kinase B (AURKB) and Aurora kinase C (AURKC) along with Raf-1 (Fig. 7A). The putative phosphatidic-acid-binding regions in the three different Aurora kinases aligned well with the Raf-1 phosphatidic-acid-binding region (A^389^-W^423^) (darker blue region towards the right of Fig. 7A). Fig. 7B,C depicts a comparison between the hydropathy plots of the phosphatidic-acid-binding regions of Raf-1 and AURA and the amino acid sequences of the phosphatidic-acid-binding region of Raf-1 and the proposed phosphatidic-acid-binding region of AURA. Hydrophobic regions that are similar between Raf-1 and AURA are depicted by the red arrows in the hydropathy graphs and are underlined in red in the amino acid sequence, whereas the basic regions that are similar between the two proteins are depicted by the green arrows in the hydropathy graphs and underlined in the corresponding amino acid sequence. Both the dark blue and light blue regions (Fig. 6A) in AURA are the amino acids most likely to bind to phosphatidic acid. Marked are hydrophobic regions and several histidine, arginine and lysine residues that could potentially accommodate both the hydrophobic side chain of phosphatidic acid, as well as its negative polar head. The region on AURA (E^171^–E^211^) has extensive sequence similarity with Raf-1 (A^389^–W^423^), which makes it a prime candidate for the binding site of phosphatidic acid to AURA.

**Fig. 8.**
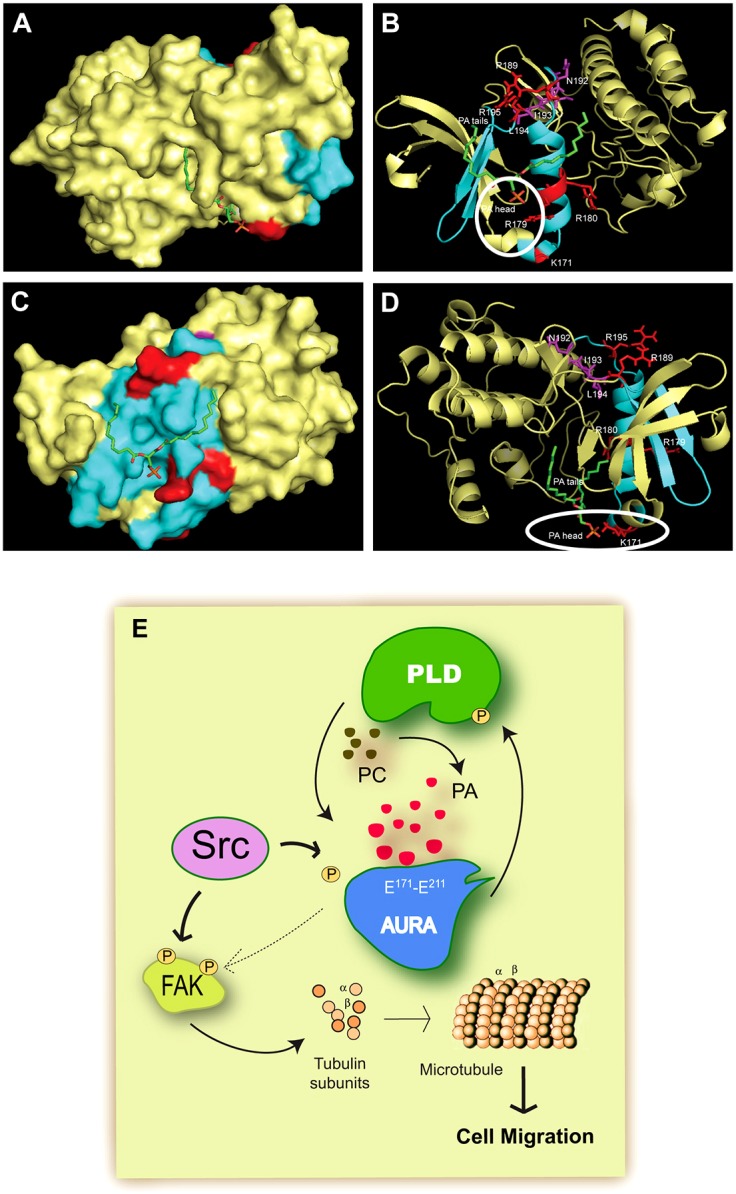
**Modeling of phosphatidic acid and AURA docking.** Solutions for the 3D structure of the AURA–phosphatidic-acid protein–lipid complex in surface (A,C) and ribbon (B,D) modes for two key amino acids: R179 (A,B) and K171 (C,D). Color code: yellow, AURA (with cyan being the alignment region considered); red, basic amino acids; magenta, hydrophobic amino acids; green, phosphatidic acid tails. Full access to the model can be found in supplementary material Table S1, which contains PDB coordinates for AURA and phosphatidic acid facing R179 and for AURA and phosphatidic acid facing K171. (E) Model of the positive interactions between AURA, PLD2 and phosphatidic acid. A reciprocal activation between PLD2 and AURA is evident from data in this study. Whereas AURA directly phosphorylates PLD2, the latter can still influence AURA by means of the release of the lipid mediator phosphatidic acid. This study also shows that phosphatidic acid binds to AURA possibly at the region indicated. AURA induces tubulin polymerization and cell migration is maximal when phosphatidic acid and Src are present.

Using three-dimensional (3D) computer-generated modeling software, we theoretically docked AURA to phosphatidic acid, which allowed us to consider many different potential binding interactions between AURA and phosphatidic acid. Multiple solutions were generated, the results of which are shown in Fig. 8A–D (full access to the model can be found in supplementary material Table S1, which contains PDB coordinates for AURA and phosphatidic acid facing R179 and for AURA and phosphatidic acid facing K171); AURA is depicted in yellow with cyan being the comparable amino acids that aligned with Raf-1, hydrophobic and basic amino acids are depicted in magenta and red, respectively, and the tails of phosphatidic acid are colored in green). We hypothesize that the phosphate head of phosphatidic acid is in close in proximity to the negatively charged basic amino acids, such as K171 (Fig. 8A,B) or R179 (Fig. 8C,D). Additionally, we propose that the hydrophobic fatty acid chains found in phosphatidic acid could interact with the hydrophobic N^192^-I^193^-L^194^ region of AURA.

## DISCUSSION

The role of AURA role during mitosis has already been defined to a large extent. The presence of diffuse staining of AURA in the cytosol and in the Golgi and perinuclear region hinted that it had a possible role unrelated to mitosis. Our study has shown for the first time that AURA ectopic expression induces a robust increase in cell migration through its positive effect on tubulin as well as tubulin polymerization. The effect of AURA on cell migration is augmented in the presence of Src and, in return, AURA also activates FAK. In addition to these two mechanisms, we report here yet a third mechanism that is important for maximal cell migration: phospholipase D and its reaction product phosphatidic acid. Phosphatidic acid is able to bind and activate AURA, causing rapid tubulin polymerization and leading to an enhanced cell migration. As shown in the model depicted in Fig. 8E, AURA phosphorylates PLD2 and increases the catalytic activity of PLD2.

Although it is known that the regulation of AURKB is mediated by the lipid raft protein flotillin-1 (Gómez et al., 2010), no studies have been conducted to date using AURA. As the data presented here indicate, binding events between AURA and phosphatidic acid occurred *in vitro* in a lipid–protein setting. The site of binding could be at the hydrophobic and hydrophilic region on AURA (E^171^–E^211^) that has extensive similitude with the Raf-1 phosphatidic-acid-binding site. Using 3D modeling, we found that the phosphate head of phosphatidic acid was in close relative proximity to the negatively charged basic amino acids (K171, R179, R180 and R195), and we propose that the hydrophobic fatty acid chains found in phosphatidic acid have the potential to interact with the hydrophobic N^192^-I^193^-L^194^ region of AURA. Our proposed model suggests that when phosphatidic acid binds to a protein, this process involves basic, as well as hydrophobic, amino acids. Therefore, we speculate that although this region on AURA (E^171^–E^211^) aligned with the phosphatidic-acid-binding region of Raf-1 (A^389^–W^423^), other basic amino acids on AURA might also be involved in phosphatidic acid binding. Further determination of the ability of phosphatidic acid to bind AURA, as determined by mutational analysis of the aligned regions would be interesting.

Although the involvement of AURA in the cell cycle and cell division is well established, a non-mitotic role for AURA in other cell signaling pathways, such as cancer cell migration and adhesion, has been recently shown (Do et al., 2014). Besides its well-defined role in mitosis, overexpression of AURA and upregulation of its enzymatic activity have been linked to tumorigenesis, specifically in ovarian, prostate, esophageal, breast and colon cancers, and inhibitory compounds have been designed to specifically inhibit AURA activity (Gritsko et al., 2003; Li et al., 2004; Tong et al., 2004; McKlveen Buschhorn et al., 2005; Baba et al., 2009). Additionally, we have shown that AURA induced tubulin polymerization and enhanced cell migration through PLD-derived phosphatidic acid.

As AURA is a target molecule for the development of anti-cancer drugs that attempt to arrest dividing cells, this very same molecules could also diminish the ability of highly metastatic cells to move away from the primary tumor. That could be accomplished with a combination of known AURA, phosphatidic acid and Src inhibitors, for possible future therapeutic approaches.

## MATERIALS AND METHODS

### Reagents

Dulbecco's modified Eagle's medium (DMEM) was from Mediatech (Manassas, VA); Opti-MEM, Lipofectamine and Plus reagent were from Invitrogen (Carlsbad, CA), [^32^P]γATP was from Perkin-Elmer (Waltham, MA); the synthetic peptide substrate for AURA (T288) (APSSRRTTLCGT) was from Bio-synthesis (Lewisville, TX), ECL reagent was from GE Healthcare (Piscataway, NJ); phosphatidic acid was from Avanti Polar Lipids (Alabaster, AL). The plasmids used in this study were as follows: pCMV6-mycDDK-AURA, pcDNA3.1-mycPLD2-WT, pEGFP-Spo20PABD (phosphatidic acid sensor) and pRK5-mycS6K-WT. The AURA inhibitor Alisertib, MLN 8237, was from Selleck Chemical (Houston, TX).

### Cell culture and transfection

COS-7 cells (ATCC, Manassas, VA) were maintained in culture at log phase using DMEM supplemented with 10% fetal calf serum and 250 mg/ml gentamicin (Sigma, St Louis, MO) at 37°C and 5% CO_2_. For transfection, appropriate plasmids were incubated with 5 µl Lipofectamine and 5 µl Plus in Opti-MEM for 15–30 min and added onto the cells. Post-transfection, cells were incubated for 36–48 h.

### Kinase assays

Either AURA or S6K was overexpressed in COS-7 cells for 36–48 h and then lysates were prepared that were used for immunoprecipitation using anti-AURA or anti-S6K antibodies conjugated to protein G agarose beads. Immunoprecipitates were incubated in the presence of 8 mM MOPS-NaOH pH. 7.0, 0.2 mM EDTA, 10 mM MgAc, 0.1 mM ATP, 1 µCi [^32^P]γATP and 100 µM of a synthetic peptide substrate specific for either AURA (T288) (APSSRRTTLCGT) or S6K (RSK2) (KKRNRTLTK) at 30°C for 10 min. Reactions were stopped by spotting onto P81 Whatman filter paper, which was washed with running water for 5 min. Filters were air-dried and cut into individual samples, placed into scintillation vials containing Scintiverse II and quantified using disintegrations per min (dpm)/mg protein.

### Immunofluorescence

Cells were transfected and plated onto glass coverslips. At 48 h post-transfection cells were fixed using 4% paraformaldehyde, permeabilized using 0.5% Triton X-100 in PBS and blocked using 10% fetal calf serum in PBS and 0.1% Triton X-100 (PBS-T). Cells were incubated with a 1∶1000 dilution of anti-Myc-FITC antibody in blocking buffer specific for Myc-tagged AURA, washed three times with PBS and then incubated in a 1∶200 dilution of anti-HA-TRITC antibody in blocking buffer specific for HA-tagged PLD2, washed again and then incubated in a 1∶2000 dilution of DAPI in PBS. Cells were washed rinsed and air dried. Coverslips were mounted onto a glass slide using Vectashield mounting medium and were then viewed using a Nikon Upright Eclipse 50i Tissue Culture Microscope, a Plan Fluor 100×/1.30 oil objective and FITC, TRITC or DAPI fluorescence filters. Photomicrographs were obtained using a Diagnostics Instrument Spot 6 digital camera and MetaVue software.

### Western blotting and co-immunoprecipitation

Wild-type PLD2 was overexpressed in COS-7 cells for 36–48 h and then lysates were prepared with Special lysis buffer (5 mM HEPES, pH 7.8, 100 µM sodium orthovanadate, and 0.1% Triton X-100) and were then subjected to SDS-PAGE and subsequent western blot analysis. Co-immunoprecipitation experiments were performed with either untransfected or AURA-transfected cells that were harvested and lysed with Special lysis buffer. Lysates were treated with 1 µl anti-AURA or anti-FLAG antibodies, respectively, and 10 µl agarose beads at 4°C and were then washed with LiCl wash buffer (2.1% LiCl, 1.6% Tris-HCl, pH 7.4) and NaCl wash buffer (0.6% NaCl, 0.16% Tris-HCl, 0.03% EDTA, pH 7.4), respectively, and sedimented at 12000 ***g*** for 1 min. Immunoprecipitated samples were analyzed by SDS-PAGE and western blot analyses for the presence of AURA and/or PLD2.

### Protein–lipid overlay assay

The method for preparing and detecting protein–lipid binding was as previously described (Dowler et al., 2002). Briefly, increasing concentrations (0, 3, 10 or 30 µg) of either 1,2-dioleoyl-sn-glycero-3-phosphate (DOPA) or 1,2-dioleoyl-sn-glycero-3-phosphocholine (DOPC) from Avanti Polar Lipids (Alabaster, AL) were spotted onto a PVDF membrane. The lipids were dissolved in a 2.0∶1.0∶0.8 ratio solution of MeOH∶CHCl_3_∶H_2_O. The membrane was blocked overnight with a 3% fatty-acid-free BSA solution, then incubated with the cell lysate at 4°C, then was washed extensively with TBS-T and incubated with anti-AURA antibody for 1–2 h at room temperature. The membrane was washed and incubated with appropriate secondary antibody and the blots were analyzed by chemiluminescence.

### Tubulin polymerization assay

Cells were either transfected with pCMV6-mycDDK-AURA for 2 days or they were treated with phosphatidic acid in 0.5% fatty-acid free BSA in PBS for 10 min prior to harvesting. An *in vitro* tubulin polymerization assay was performed as outlined by the manufacturer (Cytoskeleton, Inc., Denver, CO, USA). Cells were sonicated in tubulin lysis buffer (20 mM Tris-HCl, 20 mM NaCl, and 768 nM aprotinin) (ALB). A total of 10 µl of cell lysates were added to 85 µl of tubulin-containing buffer. Tubulin polymerization was measured for 60 min at 1-min intervals at the excitation wavelength of 340–360 nm with a bandwidth of 20 nm and the emission wavelength of 410–460 nm with a bandwidth of 10 nm in a TECAN Safire2 at room temperature.

### Enhanced cell migration assays

Post transfection, cells were resuspended at 5×10^5^ cells/ml in cell migration buffer (DMEM plus 0.5% BSA). Cells were either vehicle- or inhibitor-treated for 20 min at 37°C. A total of 200 µl was placed in the upper chambers of Transwell inserts that were separated from the lower wells by a 6.5-mm-diameter, 8-µm-pore polycarbonate membrane. EGF was diluted to 3 nM in 500 µl cell migration buffer and placed into the lower well of a 24-well plate. For cells that were inhibitor treated, inhibitor was added to the bottom well at the concentration stated in the relevant figure. Cell migration assays were incubated for 1 h at 37°C under a 5% CO_2_ atmosphere. After cell migration, inserts were discarded and the cells migrated to the bottom well were fixed for 1 h by the addition of 4% paraformaldehyde. The number of cells migrated were counted by phase-contrast microscopy of five separate fields.

### Molecular docking

The computer-simulated docking studies were performed using both HEX 6.1 and AutoDock Vina. In order to model the full-length 3D AURA structure, the HEX server (Macindoe et al., 2010) was used. The structures of diC181-PA were obtained from PDB database. HEX is a macromolecular docking program that takes into consideration both shape and electrostatic charge. In predicting molecular docking juxtapositions, it uses spherical polar fourier (SPF) to accelerate the docking calculations. The default docking control parameters of the HEX program were used to arrive at 100 docked conformations (Ritchie and Kemp, 2000). In all the HEX dockings, AURA was treated as receptor whereas the other protein partner was treated as ligand. AutoDock Vina (Trott and Olson, 2010), which allows the ligand to have flexible and rotatable bonds, was used to refine the docked structure. Docking with AutoDock Vina starts by defining a search space or binding site in a restricted region of the protein. In the present study, the receptor grid was generated using the coordinates of an active site residue N773. The resulting docking conformation was further visualized using the PyMOL (supplementary material Table S1).

### Statistical analysis

Data are presented as the mean±s.e.m. The difference between means was assessed by the single factor analysis of variance (ANOVA) test. Probability of *P*<0.05 was considered to indicate a significant difference.

## Supplementary Material

Supplementary Material
