# Supplementary material for: A non-mitotic role for Aurora kinase A as a direct activator of cell migration upon interaction with PLD, FAK and Src
Source: J Cell Sci. 2015 Feb 1;128(3):516–26. doi: 10.1242/jcs.157339 (PMC4311130; doi:10.1242/jcs.157339)
Supplement: Supplementary Material [file supp_128_3_516__index.html]

Supplementary Material 

# A non-mitotic role for Aurora kinase A as a direct activator of cell migration upon interaction with PLD, FAK and Src

## JCS157339 Supplementary Material

**Files in this Data Supplement:**

- **Supplementary Material**
