# Supplementary material for: A non-mitotic role for Aurora kinase A as a direct activator of cell migration upon interaction with PLD, FAK and Src
Source: J Cell Sci. 2015 Feb 1;128(3):516–26. doi: 10.1242/jcs.157339 (PMC4311130; doi:10.1242/jcs.157339)
Supplement: Supplementary Material [file supp_128.3.516_JCS157339.pdf]

Table S1. A non-mitotic role for Aurora kinase A as a direct activator of cell migration

Download Table S1
